# Standardizing Free-Text Data Exemplified by Age and Data-Location Fields in the Immune Epitope Database

**DOI:** 10.21203/rs.3.rs-5363542/v1

**Published:** 2024-11-14

**Authors:** Sebastian Duesing, Jason Bennett, James A. Overton, Randi Vita, Bjoern Peters

**Affiliations:** La Jolla Institute For Allergy & Immunology; La Jolla Institute For Allergy & Immunology; Knocean Inc; La Jolla Institute For Allergy & Immunology; La Jolla Institute For Allergy & Immunology

**Keywords:** Unstructured data, free-text data, data normalization, data standardization, Immune Epitope Database, ontology

## Abstract

**Background:**

While unstructured data, such as free text, constitutes a large amount of publicly available biomedical data, it is underutilized in automated analyses due to the difficulty of extracting meaning from it. Normalizing free-text data, *i.e.*, removing inessential variance, enables the use of structured vocabularies like ontologies to represent the data and allow for harmonized queries over it. This paper presents an adaptable tool for free-text normalization and an evaluation of the application of this tool to two different sets of unstructured biomedical data curated from the literature in the Immune Epitope Database (IEDB): age and data-location.

**Results:**

Free text entries for the database fields for subject age (4095 distinct values) and publication data-location (251,810 distinct values) in the IEDB were analyzed. Normalization was performed in three steps, namely character normalization, word normalization, and phrase normalization, using generalizable rules developed and applied with the tool presented in this manuscript. For the age dataset, in the character stage, the application of 21 rules resulted in 99.97% output validity; in the word stage, the application of 94 rules resulted in 98.06% output validity; and in the phrase stage, the application of 16 rules resulted in 83.81% output validity. For the data-location dataset, in the character stage, the application of 39 rules resulted in 99.99% output validity; in the word stage, the application of 187 rules resulted in 98.46% output validity; and in the phrase stage, the application of 12 rules resulted in 97.95% output validity.

**Conclusions:**

We developed a generalizable approach for normalization of free text as found in database fields with content on a specific topic. Creating and testing the rules took a one-time effort for a given field that can now be applied to data as it is being curated. The standardization achieved in two datasets tested produces significantly reduced variance in the content which enhances the findability and usability of that data, chiefly by improving search functionality and enabling linkages with formal ontologies.

## Background

A lot of data within and outside the biomedical field is unstructured, with estimates ranging as high as 95% [1]. Unstructured data is commonly underutilized due to the difficulty of automatically extracting meaningful information. In our work on the Immune Epitope Database (IEDB) [2], we have found that the unstructured data also lags behind structured data in adherence to FAIR data standards; in a 2018 analysis of the IEDB’s progress towards improved data FAIRness, an area identified for improvement was the findability of unstructured free-text data [3]. Normalizing free-text data, *i.e.*, removing variance that does not affect meaning from text, enables linkages between the unstructured data and structured vocabularies like ontologies, which can significantly improve the FAIRness and usability of the data. This paper presents a novel repository of Python scripts for free-text data normalization and an evaluation of the application of these scripts to two different sets of biomedical data from the IEDB, an age dataset and a data-location dataset.

Variance, a term that this paper uses to refer to differences in representations of information that do not change meaning, is a key problem of free-text normalization. Free-text data can contain several different kinds of variance. Character variance (such as differences in diacritic usage, whitespace, or encoding) differentiates data items like “6–8 weeks” and “6–8 weeks”. Word-level variance, which includes misspellings, abbreviations, synonyms, and colloquialisms, differentiates data items like “6–8 weeks” and “6–8 wks”. Phrase-level variance includes the ways that one idea can be expressed with different permutations of words, and it differentiates data items like “6–8 weeks” and “6 to 8 weeks”. The data items “6–8 weeks”, “6–8 weeks”, “6–8 wks”, and “6 to 8 weeks” all mean the same thing, but in their unstandardized free-text forms, they are all parsed as distinct. The aim of free-text normalization is to ensure that data items that mean the same thing look the same way. The extent to which each of those three types of variance might exist in a particular dataset is highly dependent on the nature of the data. To be broadly applicable to free-text datasets of all sorts, a free-text normalization tool must be able to address all three types of variance in a way that is flexible enough to account for different datasets’ unique normalization needs.

There is a robust history of development of automated tools for addressing some types of variance, such as spell-check technologies, but there are comparatively few holistic tools designed to normalize dataset variance at the character, word, and phrase levels. To that end, we created the free-text normalization tool ADP, which stands for Adaptable, user-Dependent, and Precise. In this paper, we examine the application of ADP to two free-text datasets from the IEDB: the age dataset and the data-location dataset, both of which were accessed using SQL queries.

The age dataset records the ages of subjects in investigations archived in the IEDB. It contains 7,151 total unique organism-age pairs (e.g., age: “6–8 weeks old”, organism name: “Mus musculus C57BL/6”), meaning some age values are duplicated in that dataset because they occur with multiple organisms; there are 4,095 unique age value strings. Strings in the age dataset typically contained one piece of information per string, and where list-like strings were present, they were legitimate lists ostensibly linked to studies that investigated subjects at multiple specific ages, e.g., the data item “21, 27 and 36 weeks”.

**Figure 1:**
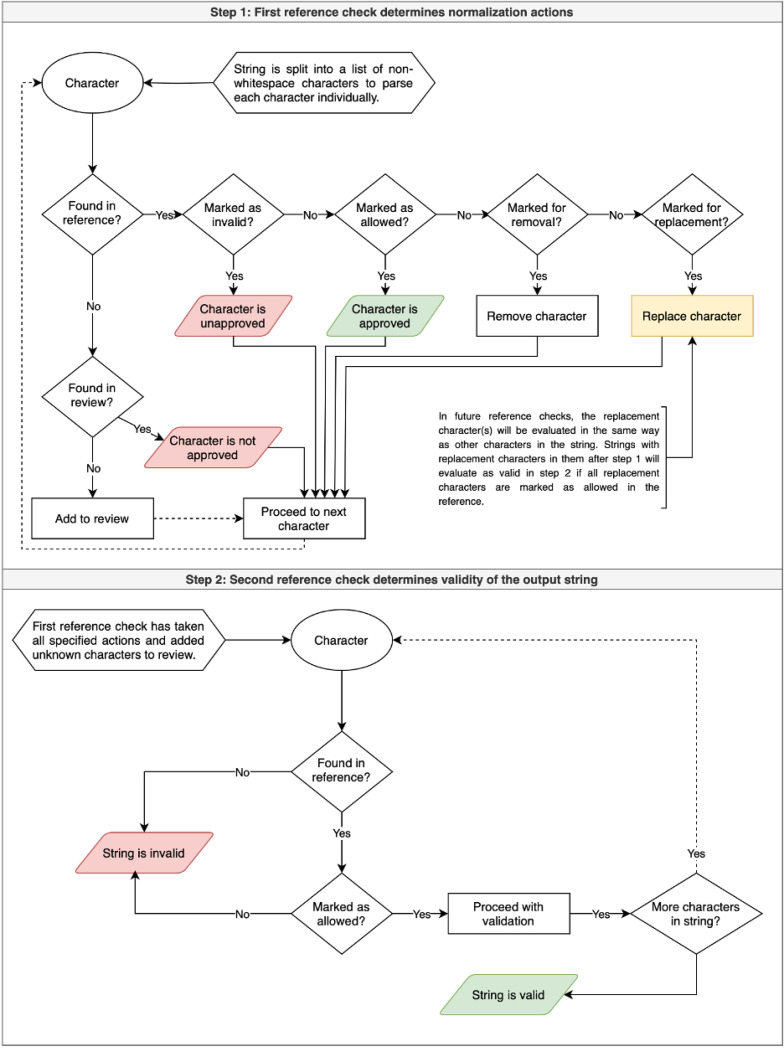
Flowchart of ADP Character and Word Normalization Processes

**Figure 2:**
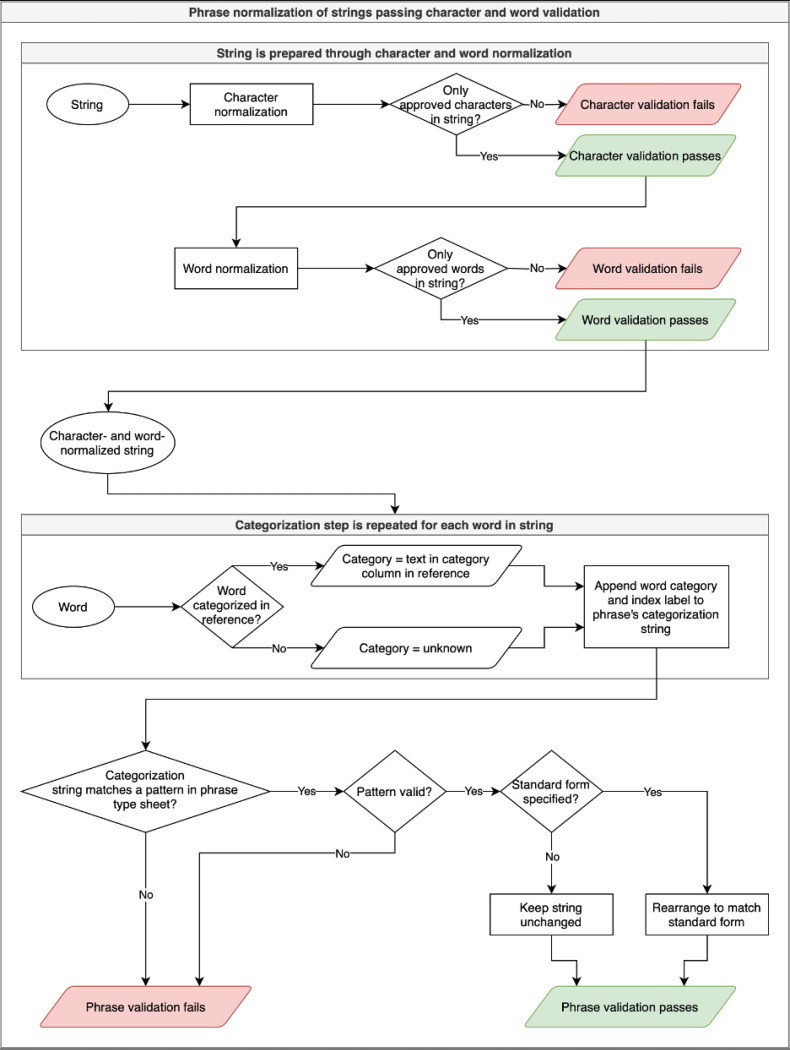
Flowchart of ADP Phrase Normalization Processes

**Figure 3:**
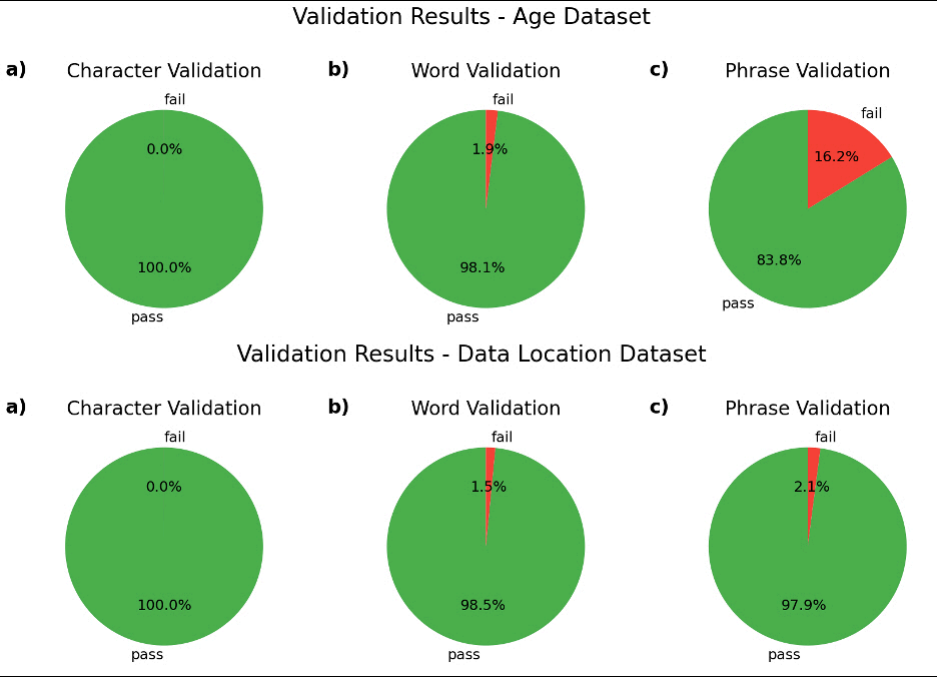
Validation Results by Dataset and Stage

**Figure 4:**
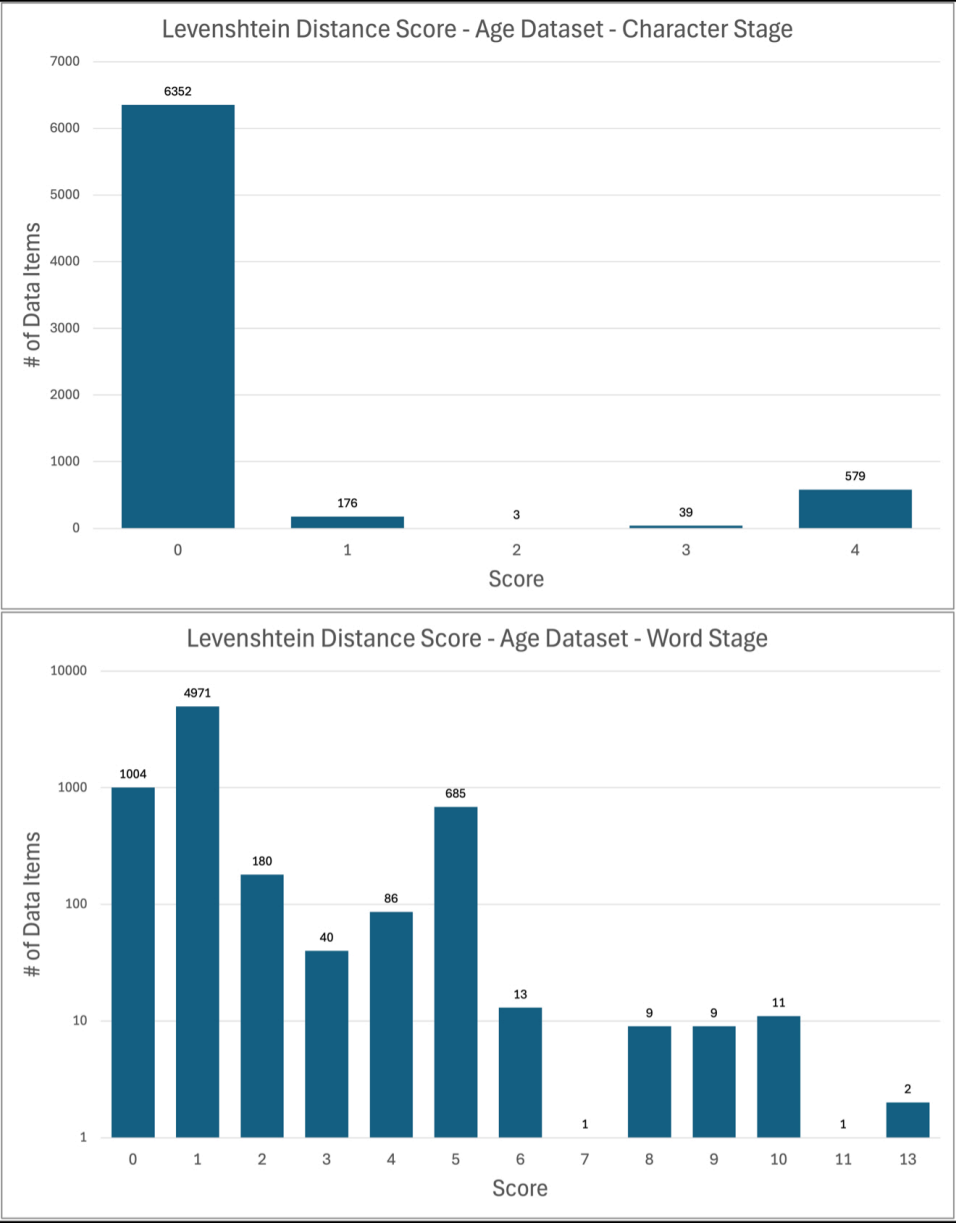
Levenshtein Distance Scores by Stage, Age Dataset

The data-location dataset records the manuscript locations in which certain data are found. It contains 251,810 unique data-location strings, such as “Cited reference [PMID: 16472860]”. In contrast with the age dataset, many strings in this dataset contained several individually valid data locations in a single line, such as “Data set S1 and S11 and Figs. 1, 2, 3, and 4”.

## Methods

ADP is a non-fully-automated normalization tool that enables a user to create standardization rules and apply them to datasets, which is available on GitHub [4]. The ADP normalization scripts are written in Python version 3.10. The core normalization scripts import the libraries os, re, and sys from the Python Standard Library and the non-native library editdistance (imported as ed). ADP is open-source software licensed under GNU GPL-3.0.

ADP’s three core normalization scripts (char_normalizer.py, word_normalizer.py, and phrase_normalizer.py) address the three types of variance outlined in the introduction: character-, word-, and phrase-level variance. At the character and word stages, ADP also logs a Levenshtein distance score for each data item to indicate the extent of the changes made in that stage. ADP uses a script (calculate_metrics.py) to pull relevant metrics from the normalized output files and generate figures using the Python libraries ast, math, matplotlib.pyplot (imported as plt), pandas (imported as pd), seaborn (imported as sns), and warnings.

### ADP Text Normalization Workflow

#### Action Decision-Based Normalization of Characters and Words

While standardizing character variance can be as simple as selecting acceptable special characters and determining case-sensitivity of the data, standardizing word-level variance involves identifying and correcting misspellings in free-text data, a process which is well-known to be “cumbersome” [5]. Normalization tools must also be able to handle “non-standard words,” including numbers, acronyms, and other abbreviations [6]. Some existing word normalization tools overcorrect and have higher rates of “unresolved errors,” or incorrectly-spelled words that the tool swaps with a context-incorrect word; others tend to undercorrect, e.g., by failing to recognize “cant” as a misspelling of “can’t” [5]. ADP uses an iterative character and word normalization process designed to prioritize accuracy of outputs.

The character and word normalization scripts share a similar rule-building workflow. When one of these two scripts is run on a dataset for the first time, it identifies distinct text units (characters or words, which for ADP’s purposes is a sequence of characters delineated by one of several common separators, like hyphens, spaces, or punctuation, or the start or end of a string) and creates a review file to be used for normalization rule-setting.

The review file is a TSV containing one row for each distinct character—except lowercase letters, digits, and a small number of basic punctuation characters, which are treated as valid for character normalization—or word found in the file. It has columns for the character or word, its context (i.e., the data item strings in which that character or word was found), and a count of its occurrences. The review file also has four action columns with the headings “replace_with”, “remove”, “invalidate”, and “allow”. Entering text in one of the action columns (which we refer to as “making an action decision”) sets a rule for the behavior of the script concerning the character or word in that row during future runs of the script. Table 2 describes how entering text in one of the action columns modifies the behavior of the script.

Every time the script is rerun, it moves any review file rows in which an action decision has been made to a reference file, which serves as a bank of rules for the behavior of the script.

Tables 3 and 4 contain examples of the rules applied to these datasets at the character and word stages. To see all normalization rules applied at the character and word stages, please refer to the reference files in the ADP repository [4].

Following the transfer of rows with new action decisions from the review file to the reference file, the script runs its normalization functions, applying the rules based on the user’s action decisions to the dataset, and it checks for any new text units that do not have a line in either the review file or the reference. See Fig. 1 for a visual representation of how this process works during the character normalization stage.

In the character normalization stage, data items pass validation if in the second reference check (as shown in the diagram), only allowed characters are found in the string; otherwise, validation fails. Only data items that pass character-level validation are normalized in the word normalization stage. Data items pass word-level validation if in the second reference check, only allowed words are found in the string; otherwise, validation fails.

### Pattern-Based Normalization of Phrases

ADP phrase normalization uses a process of matching phrase structures to user-defined patterns. This process begins in the word normalization stage. In the word review and reference TSV, there is an additional “category” column. Adding text to this column in the row of a particular word asserts the category to which that word belongs, *e.g.*, in rows for the words “week”, “month”, and “year”, the category has been set to “unit” in the word reference TSV for the age dataset.

When the phrase normalization script is called, it divides the data item into individual words as was done for the word normalization phase. The script tracks the word’s place in the string and any delimiters (including punctuation, whitespace, and the start or end of a string) on either side of the word. Then, it searches the word reference file to see if a category has been assigned to the word; if not, it categorizes the word as “unknown”. The script produces a string that uses a simple grammar to indicate the categories of each word and their position in the string, e.g., the age datum “6 week mean” is parsed as “[number(0)][unit(1)][statistical(2)]”. The phrase categorization string is stored in a dedicated column in the phrase normalization output file to enable the user to determine which phrase structures occur the most frequently in a dataset and develop normalization rules accordingly.

Like the character and word normalization phases, the phrase normalization phase depends on the user to create rules for distinct phrase structures. A dataset’s phrase-type ruleset (found in age_phrase_types.tsv and data_loc_phrase_types.tsv) establishes a name for a pattern, indicates whether or not it is a valid pattern (e.g., in the age dataset, a data item consisting of a number and a unit is valid, but a number by itself is not, as being unitless makes its meaning uncertain), and sets a rule for how phrases that match that pattern should be formatted. See Table 5 for examples.

The categorization string, e.g., [number(0)][unit(1)][statistical(2)] (extracted from “6 week mean”), is matched to a pattern—in this case, the pattern called “statistical”—which matches to the structures of data items that provide a mean or median age value. In the “standard_form” column in the phrase-type ruleset, the user can specify how data items matching a pattern should be formatted. In the case of “6 week mean”, the standard form is represented as “[2]: [0] [1]”, in which the numbers in brackets refer to the indices from the categorization string, and how they should be arranged within the standard form string.

The phrase normalization script generates a blank phrase-type ruleset file if none exists, but if one exists, it checks each data item’s categorization string against any patterns in the file and applies the pattern in the “standard_form” column if applicable by inserting words where their indices are placed in the standard form string. Through this process, “6 week mean” is rearranged to match the standard form string “[2]: [0] [1]”, so the output for that data item is “mean: 6 week”. This workflow ensures that data items with diverse structures, like “6 week mean” and “mean = 6 week”, take on a single standard phrase structure, like “mean: 6 week”. The specific structure we chose for data items of this type is arbitrary; the crucial part is the ability to quickly modify diversely expressed data items into one standard style.

Table 5 below contains sample rows from both datasets’ phrase type tables as examples of the rules applied to these datasets. To see all normalization rules applied at the phrase stage, please refer to the phrase type files in the ADP repository [4].

Only data items passing validation at the character and word stages are normalized at the phrase stage. Data items pass phrase-level validation if they match a pattern designated as valid in the phrase-type ruleset. Otherwise, validation fails. The phrase normalization and validation processes are visualized in the flowchart below.

### Measuring String Change During Normalization

The ADP normalization code imports the package editdistance to measure the Levenshtein distance between the inputs and outputs in the character and word normalization stages. The normalized output files dedicated columns for distance scores comparing the character-normalized string against the original and the word-normalized string against the character-normalized string. Due to the phrase normalization stage often involving changes in word order, Levenshtein distance ceases to be a sensible measure of continuity between input and output at the phrase normalization stage.

### Modular Normalization & Accessory Stages

The ADP normalization process is designed to be modular; because it is split into discrete processes for character, word, and phrase normalization, it is possible to plug in accessory stages to address dataset-specific normalization needs that are not easily handled within the pre-defined stages. The data-location dataset, for instance, implements an accessory stage to split list-like data items into individual strings for data location.

### Data-Location Splitting

Because the data-location dataset contained list-like data items in which several distinct data locations were included in a single data item (e.g., the real data item “Fig. 2A,B,C, Fig. 6.”), phrase normalization would be much more difficult without splitting list-like inputs into multiple items that could then be normalized independently. The script functions as a pre-phrase-normalization stage for the data-location dataset; that script creates multiple rows from list-like data items, transforming the single data item “Fig. 2A,B,C, Fig. 6.” into a set of segments including “figure 2a”, “figure 2b”, “figure 2c”, and “figure 6”. Each segment is separated into a distinct row, which is assigned a post-splitting index and an original index to be able to both track segments individually and trace them back to the list-like data items from which they were originally split.

When phrase normalization is applied to the data-location dataset, because the segments have been split into their own rows, they are treated as distinct phrases, allowing all of the “figure *x*” example segments above to match to a single pattern, rather than needing dedicated patterns to match to each list-like permutation.

### Sample Normalized Data Items

Table 6 contains sample data items from the age and data-location datasets. The columns represent the progression of these data items through the normalization process, with changes made by the character, word, and phrase normalization parts of the code represented in those respective columns. Note that for the data-location dataset, the list-like phrase-normalized strings are split into individual TSV rows for each data item in the list, e.g., the single input data item “Fig. 2A,B,C, Fig. 6.” becomes four output data items: “figure 2a”, “figure 2b”, “figure 2c”, and “figure 6”.

## Results

Using ADP’s normalization scripts on the IEDB age and data-location datasets demonstrates that it is possible to use ADP to effect significant improvements to the overall standardization of a dataset.

### User Action Efficiency

ADP is a tool for the development and implementation of standardization rules. Accordingly, the thoroughness with which a user makes action decisions (in the character and word stages) and builds phrase type patterns (in the phrase stage) determines the overall success of ADP at standardizing a dataset. The data presented in this manuscript is the result of a non-exhaustive approach to both datasets in which rule-setting for particularly common characters, words, and phrases was prioritized, to represent a practical and realistic normalization outcome.

Table 7 provides an overview of the extent of the normalization rule-setting done for each dataset. The “items in review” counts reflect the number of characters or words for which action decisions were not made at the time of manuscript submission. The “items in reference” counts reflect the number of characters or words for which action decisions were made. The “phrase-type patterns” counts reflect the number of user-generated patterns against which phrases are matched to determine their validity, and “valid phrase-type patterns” reflect how many of the defined patterns are specified as valid phrases.

The results presented in this manuscript are accordingly the results of a fairly conservative rule-setting effort intended to prioritize the creation of rules targeting high-occurrence characters, words, and phrase patterns. More comprehensive normalization and higher validity rates at each stage could be achieved by targeting increasingly lower-frequency characters, words, and phrases. Ultimately, reasonable stopping points will vary for each dataset; making action decisions and creating phrase patterns for increasingly infrequent characters, words, and phrases offers diminishing returns in overall dataset standardization.

### Validity Rates by Dataset and Stage

ADP validates data items at each stage. In the character stage, data items pass validation if they contain only characters that have been marked as allowed. Data items pass validation at the word stage if they contain only words that have been marked as allowed. In the phrase stage, data items pass validation if they match to a pattern designated as valid. The word and phrase stages only attempt to normalize data items that have passed validation in the previous stage(s). The Validation Results by Dataset and Stage figures below show the rates of validity achieved with the aforementioned non-exhaustive rule-setting approach.

In the character stage, validity rates for both datasets are above 99%. These character validation results were achieved following 21 action decisions for the age dataset and 39 action decisions for the data-location dataset in the character normalization stage (see Table 7).

In the word stage, validity rates for both data sets are above 98%. These word validation results were achieved following 94 action decisions for the age dataset and 187 action decisions^4^ for the data-location dataset in the character normalization stage (see Table 7).

The age dataset’s validity rate at the phrase stage is significantly lower than that of the data-location dataset: 83.8% of data items pass phrase validation in the age dataset, while 97.9% of data items pass phrase validation in the data-location dataset. This is the result of a relatively large number of data items that match invalid patterns. In particular, for the age dataset, numerical exact values (e.g., “7”) and ranges without units (e.g., “8–10”) are designated as invalid phrase types because that age dataset contains ages expressed in hours, days, weeks, months, and years, so any number-unit combination is theoretically possible; without a unit, numerical age values are practically meaningless. As is recorded in the phrase-normalized age dataset file, of the 1019 data items that failed phrase validation, only 105 (1.47% of all data items) failed because they did not match any pattern; all the rest failed because they matched a pattern designated as invalid.

These phrase validation results were achieved by matching against 16 phrase-type patterns for the age dataset and 12 patterns for the data-location dataset (see Table 7).

It is evident that a relatively low number of user action decisions is sufficient to produce very high rates of validity in at least these two free-text datasets. Notably, in both the character and word stages, reaching similar results (> 99% validity in the character stage and > 98% validity in the word stage) in the two datasets required only about twice as many action decisions in the data-location dataset as in the age dataset, despite that the former dataset is more than 35 times longer than the latter.

### Extent of Change to Data Items

**Figure 5:**
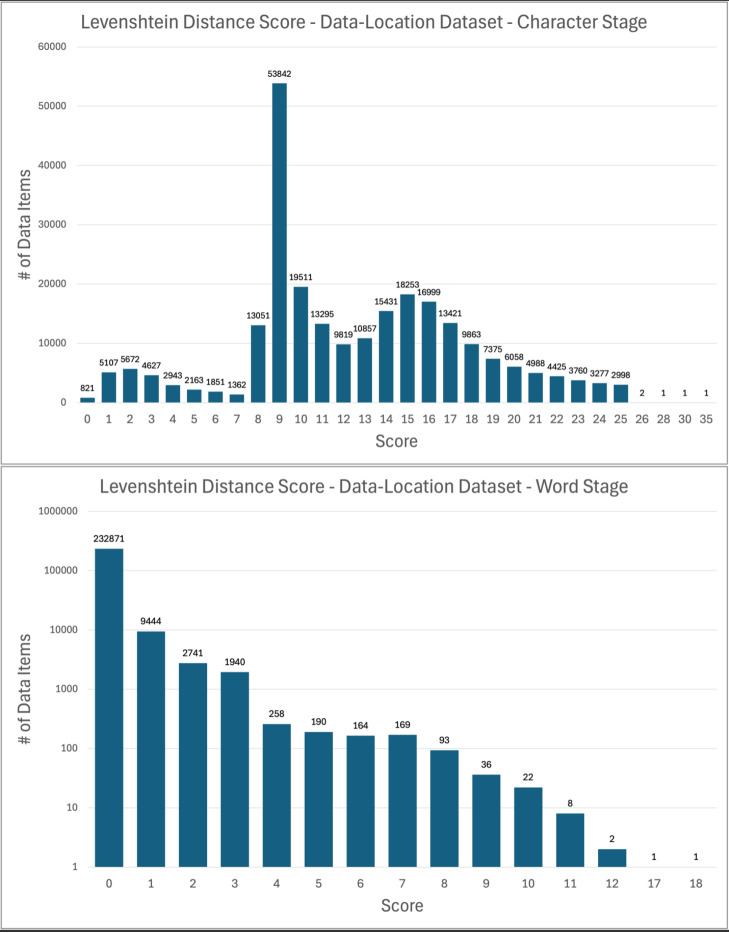
Levenshtein Distance Scores by Stage, Data-Location Dataset

In the character and word stages, the values in the Levenshtein distance score columns (see Measuring String Change During Normalization above) serve as indicators of the extent to which strings are modified during the normalization process. Figures 4 and 5 show the frequency distributions of Levenshtein distance scores by dataset and stage. Note that the word stage figures for both datasets use a logarithmic scale for clarity.

For the age dataset, Levenshtein distance score frequency graphs show that most data items receive little modification during the character and word stages. The notable spike at a score of 1 in the word stage results from the abundance of age data items with plural units that were normalized to singular; the score of 1 frequently represents the removal of an “s” from “years”, “months”, or “weeks.”

In the data-location dataset, the uniform nature of much of the dataset (namely the > 200,000 lines of HLA Ligand Atlas URLs) produces other spikes in the character stage Levenshtein distance frequency chart. The spike at 9 is one such case. Of the 57,517 data-location data items with a Levenshtein distance score of 9 at the character stage, 91% (52,522) are HLA Ligand Atlas URLs that have paths that a string of 9 uppercase letters (e.g., “https://hla-ligand-atlas.org/peptide/AAAAAQSVY”). The URLs resolve in the same way with lowercase and uppercase letters in that path; the former URL is functionally equivalent to “https://hla-ligand-atlas.org/peptide/aaaaaqsvy”, so normalizing to lowercase does not result in any lost meaning. Levenshtein distance scores at the word stage cluster strongly around 0 for the data-location dataset, a reflection of the fact that a large portion of the dataset, namely the URLs, received no word normalization.

Levenshtein distance ceases to be a useful metric at the phrase stage, at which it is often desirable to make significant changes to the overall structure of the data item. Straightforward and benign changes like alterations in word order produce high Levenshtein distances. Accordingly, Levenshtein distance scores are not tracked at the phrase stage.

### Data-Location Phrase Splitting and Phrase-Part Validity

Because the data-location dataset included a high number of list-like inputs made up of several individual data locations, the data items in that dataset were put through a splitter script that divided list-like data items so that each output datum referenced exactly one data location (see Data-Location Splitting above).

For this dataset, we calculate additional relevant metrics. Split phrase count (listed in the split_phrase_count column) refers to the total number of outputs split out of an original input data item; e.g., the input item “Table 1 and Fig. 1”, which is split into the data items “Table 1” and “figure 1”, has a split phrase count of 2. Validity rate is the number of valid output data items divided by the split phrase count. A validity rate of 1 means that every output data item that derives from a particular input data item is valid, while a validity rate of 0 means that none of those output data items are valid. Split phrase count and validity rate (along with all other analytics, like Levenshtein distance scores) are recorded in the phrase-normalized output file exactly once for each input data item so that means and frequency distributions of those metrics are not skewed by the row-count increase that occurs during phrase splitting.

As is evident in Fig. 6, the large number of HLA Ligand Atlas URLs in the dataset concentrate both the split phrase count and validity score around 1, as the URLs are all unsplit and valid. Including URLs, the mean split phrase count is 1.24 (standard deviation 0.92), and the mean phrase validity rate is 1.00 (standard deviation 0.06).

It is noteworthy that the data items with high split phrase counts tend towards high validity rates. It appears that those data items tend to be simple and orderly lists, such as the data item “Figures 1, 2, 3, 4, Supplementary Figs. 2, 3, 4, 5, 6, 7, 8, 9, 10, 11, 12, 13”, which has a split phrase count of 16 and a validity rate of 1.0. Such data items are simpler to split, and their split outputs are individually simpler and more readily matchable to basic phrase patterns than the less uniform lists that occur towards the middle of the split phrase count range, such as “Table 4 and Figs. 1 and 2 and Supporting Information S2 Figure” (split phrase count 4, validity rate 0.75).

When examining only non-URL data items, strong clustering around a validity rate of 1 remains, but with a more obvious spread of split phrase count values, as is evident in Fig. 7. Excluding URLs, the mean split phrase count is 3.19 (standard deviation 1.91), and the mean phrase validity rate is 0.96 (standard deviation 0.17).

The implementation of data-location phrase splitting achieves very high rates of validity even among the complicated minority made up of non-URL data items.

## Discussion

### Measuring Normalization Empirically

The ADP toolset provides several metrics by which a user can measure the extent to which ADP normalization modifies the data, including Levenshtein distance scoring and validation pass/fail rates. These metrics are intended to approximate the degree to which the ADP normalization code improved the overall normality of the data without losing the original string’s meaning. However, empirically evaluating the success of the normalization process as a whole remains difficult due to the lack of a clear universal metric for dataset normalization. A useful future direction would be to establish an empirical way to measure degrees of standardization in unstructured datasets; ideally, such a metric would allow comparisons between free-text datasets’ spelling accuracy, adherence to grammar, and stylistic consistency.

### Evaluating the Utility of ADP

While the age and data-location datasets are distinct enough in size, content, and style to make a case for the flexibility of the ADP rule-setting framework for normalization, its use on these two datasets is not sufficient to demonstrate that ADP is a useful tool for a truly wide range of free-text datasets. Further experimentation with other free-text datasets will be necessary to ensure that ADP normalization is adaptable enough to be used with a diverse range of free-text datasets.

Developing frameworks for testing the accuracy of ADP’s outputs compared to other normalization methods is an active priority. ADP’s user-dependence is a design feature that was implemented specifically because we hypothesize that it will result in higher precision of normalization results compared to predictive normalization tools, which can struggle with certain context-specific normalization decisions, like handling instances of “cant” occurring as a synonym of “slang” rather than a misspelling of “can’t”, that humans can make quickly and accurately [5]. Future testing will likely include evaluating how effectively ADP normalization preserves the meaning of data items throughout the normalization process compared to analogous normalization tools.

Some recent tools for free-text standardization make use of large language models to perform standardization tasks. One such tool is CleanAgent, which uses an LLM agent to identify the types of data (e.g., phone number, email address, date) in each column of a CSV, write and run Python code to standardize each column’s data based on its type, and interact with the user throughout the standardization process [7]. CleanAgent’s “hands-off” approach to data standardization is intended to ensure ease of use and efficiency, as CleanAgent is designed to enable “data scientists to input their standardization requirements in one instance” [7]. At the time of the writing of this manuscript, the authors were unable to run CleanAgent on either the age or data-location datasets. We have reached out to the developers of CleanAgent about a recurring error message; we aim to do a direct comparison between the free-text normalization outcomes from CleanAgent and ADP in the future. The following analysis of CleanAgent’s utility is accordingly based solely on the contents of the CleanAgent demonstration publication [7].

CleanAgent’s column type identification processes appear to be somewhat limited. As shown in its 2024 demonstrational publication, CleanAgent’s column type identifier attributes the datatype “address” to the “Name” column of its test CSV, which stands out as a potential misidentification; the dataset used in that demonstration is not included with that paper nor does it appear to be listed in the GitHub repository for CleanAgent [8], so we were unable to verify if that is indeed a valid datatype for that column. Furthermore, as shown in that demonstration publication, CleanAgent did not identify a datatype for the columns named “AGE” and “weight ” [7], suggesting that CleanAgent may struggle to standardize columns of data that, unlike email addresses or phone numbers, lack a well-defined standard form. CleanAgent’s LLM-based normalization appears to be an efficient solution to the problem of standardizing fairly simple data items (e.g., dates or contact information), and it is designed to accomplish normalization with very little work on the user’s part. ADP, by comparison, requires more of the user’s time and effort but handles more complex and widely-varying data items.

LLM-based normalization tools are limited by the accuracy of the LLMs on which they depend. Output accuracy is a vitally important feature of standardization processes for biomedical datasets. Ostensibly, LLM accuracy will continue to improve, but in the meantime, there remains a need for high-precision standardization tools for use on datasets that require extremely high accuracy on free-text fields that are often far messier than comparatively simple “date” or “phone number” fields. ADP is designed for this niche. The authors intend to conduct further empirical evaluation of the accuracy of ADP and comparable LLM-based normalization tools.

### Productive Value of Results

These datasets contain all distinct age and data-location values recorded in the IEDB, but many of the individual values in these datasets represent thousands of instances of that value. As a result, the effect of normalizing these datasets is multiplied. The IEDB records more than 18 million total age values and more than 21 million total data-location values [4], so applying normalization to these data values in the IEDB will accordingly produce significant improvements to findability and usability of millions of lines of data.

### Improving Data Findability

Using the ADP normalization toolkit, we normalized the age and data-location free-text datasets from the IEDB, two datasets with very different content and normalization needs, in such a way that renders the data in these datasets searchable, findable, and ontologizable in a way that they simply were not before. Standardizing the text in these datasets will enable the forthcoming implementation of dedicated search tools for these datasets in the IEDB. For instance, IEDB users could query for data from experiments on mice less than 28 days old and receive results within that range including ages originally expressed with varying formats and units (e.g., ‘10–20 days old’, ‘2 days’, ‘24 h’, ‘1–3 wks’). Similarly, by ontologizing categorical age data items like ‘juvenile’, ‘calf’, ‘foal’, ‘piglet’, or ‘child’, we can enable searches for pre-adult life stages across species.

The FAIR data principles identify searchability (principle F4) as a critical aspect of data findability, so improving the IEDB’s search functionalities is core to the IEDB’s effort to improve its overall data FAIRness [9]. The data-location dataset in particular was identified as a promising candidate for work to improve the IEDB’s FAIRness in a 2018 analysis of the IEDB’s adherence to the FAIR standards [3].

Accordingly, the normalization performed on the data-location dataset using ADP completes that long-standing goal and demonstrates the IEDB’s ongoing commitment to improving data FAIRness in immunology.

### Enabling Ontologization of Free-Text Data

By standardizing the characters, words, and phrase structures in free-text datasets, ADP makes it easier to ontologize those datasets. Several prior publications have illustrated the benefits of linkages between IEDB data and formal ontologies [2,3,10,11]. Already, many IEDB data fields are mapped to terms from a wide range of ontologies, such as the “Organism” field being mapped to NCBI Taxonomy [12] terms and the “Evidence Code” field being mapped to Evidence Ontology [13] terms [14]. By standardizing the terms in use in the age and data-location datasets, ADP normalization is an effective step towards ontologizing the data in these fields. In particular, promising next steps include the ontologization of units in the age dataset via the Unit Ontology [15] and document parts via the Information Artifact Ontology and Ontology for Biomedical Investigations [16]. Should ADP prove effective on other free-text datasets within and beyond the IEDB, it will make it possible to reap the benefits of ontologization from large amounts of previously underutilized biomedical data.

## Conclusions

While further testing is necessary to validate ADP normalization on other datasets, preliminary evaluations of its application to the age and data-location datasets suggest that ADP normalization can produce high rates of output validity in diverse free-text datasets following a relatively low number of user action decisions.

The Immune Epitope Database (IEDB) has made significant efforts over the past several years to improve its adherence to FAIR data standards through improvements to findability and interoperability of its data. Creating linkages with formal ontologies is a pillar of the IEDB’s efforts to improve interoperability, but these efforts have been concentrated on standardized datasets. The ability to standardize free-text datasets would enable further FAIRness and more effective utilization of the vast quantities of free-text data in the IEDB. Our preliminary results are promising indications that ADP normalization can standardize free-text datasets efficiently and accurately.

## Figures and Tables

**Table 1:** Example Age & Data-Location Data Items

Age Dataset	Data-Location Dataset
6 to 8 weeks	Figures 2, 3, 4, 5, 6, S4, S5, S7, Tables 2, 3, 4 and 5
Adults (pregnant)	PDB: 5EC1, 5EC2, 5EBW, 5EBL, 5EBM
Mean age of 32.2 years with a range from 18 to 49 years	Richardson et al. Virol 1986;155:508–523 [PMID: 3788062]
18–22 months or 4–6 months	pg. 1410 and J. Virol. 61:1358–1367

**Table 2:** Action Decisions

Action Column	Function
replace_with	This character or word is replaced with the text that is entered in this column.
remove	This character or word is removed from the data items in which it occurs.
invalidate	This character or word remains as-is, and data items containing this character or word will fail validation.
allow	This character or word remains as-is, and this character or word is considered an accepted text unit for validation.

**Table 3:** Sample Character Normalization Rules & Applications to Data Items

Dataset	Char.	Occurrences	Example string	Rule	Post-normalization string
age	=	65	“mean age = 30 years”	Allow	“mean age = 30 years”
age	–	31	“20–67 years”	Replace with: -	“20–67 years”
data-loc	&	53	“Abstract & p. 664”	Replace with: and	“abstract and p. 664”
data-loc	€	10	“Figure 1 and Fig. 1â€”figure supplement 1 and PDB 6HD8”	Invalidate	Invalid, not normalized

**Table 4:** Sample Word Normalization Rules & Applications to Data Items

Dataset	Word	Occurrences	Example string	Rule	Post-normalization string
age	old	710	“6–10 week old”	Remove	“6–10 week”
age	wk	57	“8–10 wk”	Replace with: week	“8–10 week”
data-loc	fig	285	“Figs. 1 and 2”	Replace with: figure	“figure 1 and 2”
data-loc	file	148	“additional file 1”	Allow	“additional file 1”

**Table 5:** Sample Phrase Normalization Rules

Dataset	Pattern name	Pattern	Standard form	Example matched phrases	Example normalized phrases

age	range	[number(0)]	[0]-[2] [3]	“6 to 8-week”,	“6–8 week”,
		[range_indicator(1)]			
		[number(2)] [unit(3)]		“44.9 to 74.1	“44.9–74.1
				year”,	year”,
				“36 to 68.2	“36–68.2 year”
				year”	

age	statistical	[statistical(0)] [number(1)] [unit(2)]	[0]: [1] [2]	“mean 29.8 year”,	“mean: 29.8 year”,
				“mean: 30 year”,	“mean: 30 year”,
				“median : 7.5 year”	“median: 7.5 year”

data-loc	pdb id	[pdb(0)] [pdb_id(1)]	[0] [1]	“pdb 1mfd”, “pdb 1rzj”, “pdb 1rzk”	“pdb 1mfd”, “pdb 1rzj”, “pdb 1rzk”

data-loc	loc number	[location(0)] [number(1)]	[0] [1]	“page 11782”, “information 9”, “data 1”	“page 11782”, “information 9”, “data 1”

**Table 6:** Sample Data Items at Each Stage

Dataset	Before Normalization	Character Normalized	Word Normalized	Phrase Normalized
Age	Six week old	six week old	6 week	6 week
Age	6–8 week	6 to 8-week old	6 to 8-week	6–8 week
Age	Median age 6.3 years	median age 6.3 years	median 6.3 year	median: 6.3 year
Data-Location	Additional File 4, Tables 1 and 2	additional file 4, Tables 1 and 2	additional file 4, Table 1 and 2	[‘additional file 4’, ‘Table 1‘, ‘Table 2‘]
Data-Location	Figure 2A,B,C, Fig. 6.	Figure 2a,b,c, Fig. 6	figure 2a,b,c, Fig. 6	[‘figure 2a’, ‘figure 2b’, ‘figure 2c’, ‘figure 6’]
Data-Location	Figure 2A,B, Suppl Fig. 2	figure 2a,b, suppl Fig. 2	figure 2a,b, supplemental Fig. 2	[‘figure 2a’, ‘figure 2b’, ‘supplemental Fig. 2’]

**Table 7:** Number of Action Decisions by Dataset

	Age Dataset	Data-Location Dataset
Characters in review	1	7
Words in review	84	1160
Characters in reference	21	39
Words in reference	94	5780 counting mass-allowed Protein Data Bank (PDB) identifiers, otherwise 186^3^
Phrase-type patterns	16	12
Valid phrase-type patterns	9	11

## Data Availability

All code and data discussed in this manuscript is available in the following GitHub repository: https://github.com/sebastianduesing/adp

## Footnotes

3. The data-location dataset contained a large number of Protein Data Bank (PDB) identifiers that parsed as distinct words. These IDs, which follow a standard four-character alphanumeric format, were selected using a regular expression and then mass-allowed. There are 186 non-PDB-ID words in the data-location reference file.

4. 186 row-by-row action decisions plus one mass-allow of Protein Data Bank (PDB) identifiers, as described in footnote 1, performed via regular expression selection of the rows containing PDB identifiers.
